# MicroRNA Profiling in Prostate Cancer - The Diagnostic Potential of Urinary miR-205 and miR-214

**DOI:** 10.1371/journal.pone.0076994

**Published:** 2013-10-22

**Authors:** Anvesha Srivastava, Helle Goldberger, Alexander Dimtchev, Malathi Ramalinga, Juliet Chijioke, Catalin Marian, Eric K. Oermann, Sunghae Uhm, Joy S. Kim, Leonard N. Chen, Xin Li, Deborah L. Berry, Bhaskar V. S. Kallakury, Subhash C. Chauhan, Sean P. Collins, Simeng Suy, Deepak Kumar

**Affiliations:** 1 Cancer Research Laboratory, Department of Biology, Chemistry and Physics, University of the District of Columbia, Washington, D. C., United States of America; 2 Biochemistry Department, Victor Babes University of Medicine and Pharmacy Timisoara, Romania; 3 Lombardi Comprehensive Cancer Center, Georgetown University, Washington, D. C., United States of America; 4 University of Tennessee Health Science Center, Memphis, Tennessee, United States of America; The University of Kansas Medical center, United States of America

## Abstract

Prostate cancer (PCa) is the most common type of cancer in men in the United States, which disproportionately affects African American descents. While metastasis is the most common cause of death among PCa patients, no specific markers have been assigned to severity and ethnic biasness of the disease. MicroRNAs represent a promising new class of biomarkers owing to their inherent stability and resilience. In the present study, we investigated potential miRNAs that can be used as biomarkers and/or therapeutic targets and can provide insight into the severity and ethnic biasness of PCa. PCR array was performed in FFPE PCa tissues (5 Caucasian American and 5 African American) and selected differentially expressed miRNAs were validated by qRT-PCR, in 40 (15 CA and 25 AA) paired PCa and adjacent normal tissues. Significantly deregulated miRNAs were also analyzed in urine samples to explore their potential as non-invasive biomarker for PCa. Out of 8 miRNAs selected for validation from PCR array data, miR-205 (*p*<0.0001), mir-214 (*p*<0.0001), miR-221(*p*<0.001) and miR-99b (*p*<0.0001) were significantly downregulated in PCa tissues. ROC curve shows that all four miRNAs successfully discriminated between PCa and adjacent normal tissues. MiR-99b showed significant down regulation (*p*<0.01) in AA PCa tissues as compared to CA PCa tissues and might be related to the aggressiveness associated with AA population. In urine, miR-205 (*p*<0.05) and miR-214 (*p*<0.05) were significantly downregulated in PCa patients and can discriminate PCa patients from healthy individuals with 89% sensitivity and 80% specificity. In conclusion, present study showed that miR-205 and miR-214 are downregulated in PCa and may serve as potential non-invasive molecular biomarker for PCa.

## Introduction

Prostate cancer (PCa) is the most frequently diagnosed male cancer and the second-leading cause of oncological mortality in men in the United States [1]. In 2013, ∼238,590 new cases of PCa are estimated to be diagnosed in the USA which can claim ∼29,720 deaths [1]. African American (AA) men are disproportionately affected with PCa with an incidence rate two-third higher and mortality rate twice as high when compared to Caucasian Americans (CA) [2]. Owing to its non- specific symptoms and gradual progression, PCa is generally diagnosed at an advanced stage. However, if diagnosed at an early stage PCa can be treated successfully.

Routinely performed tests for early detection of PCa include digital rectal examination (DREs) and prostate-specific antigen (PSA) testing. PSA testing is non-specific, as elevated PSA levels due to benign prostatic hyperplasia (BPH), infection, and/or chronic inflammation may lead to confounding outcomes [3]–[4]. Low specificity (PSA testing) and low sensitivity (DRE) of these tests restricts their diagnostic significance [5]. Although clinical trials advocate that PSA screening greatly facilitates the early diagnosis of PCa, whether this screening procedure significantly lowers the PCa mortality remains a question of debate [6]–[7]. A recent meta-analysis concluded that routine screening with either a DRE or PSA offers no benefit and does not influence PCa mortality [8]. To overcome these drawbacks, additional biomarkers have been proposed including PSA derivatives such as total PSA velocity (total PSAV) and different molecular forms of PSA such as free PSA, BPSA, pro-PSA, and intact PSA [9]. Other blood based biomarkers such as human glandular kallikrein 2 (hK2), urokinase plasminogen activator (uPA) and its receptor (uPAR), transforming growth factor-beta 1 (TGF- β1); interleukin-6 (IL-6) and its receptor (IL-6R) have been studied alone or in combination with PSA and suggested for diagnosis, staging, prognostics, and monitoring of prostate cancer [10]. However, due to the heterogeneous nature of this disease, additional prognostic biomarkers are urgently needed for better prediction of disease progression that can help in clinical decision making about the timing of biopsy and necessity of treatment.

MicroRNAs (miRNAs) are small (18 to 24 nucleotides), highly conserved, non-coding RNA molecules that regulate gene expression post-transcriptionally [11]–[12]. Computational studies suggest that over 60% of mammalian gene transcripts are regulated by miRNAs [13]–[14]. MiRNAs play key regulatory role in divergent cellular processes, including cell cycle, proliferation, differentiation, and apoptosis [15]. Recent studies have implicated various miRNAs in the development and progression of multiple human cancers and also as potential biomarker in cancer diagnosis and prognosis [16]–[19].

Considering the heterogeneous nature of cancer tissues, laser capture micro dissection (LCM) offers an attractive approach to isolate defined cell populations from FFPE specimens [20]. LCM in combination with qRT-PCR technology has been utilized for accurate measurement of gene expressions from FFPE samples. In the present study, we utilized LCM and performed miRNA profiling in prostate cancer and corresponding adjacent normal tissues to identify miRNAs associated with the development of PCa. The miRNAs identified were further validated in larger sample sets. To test their non-invasive diagnostic potential, we also evaluated the expression of candidate miRNAs in an independent set of urine samples from PCa patients.

## Materials and Methods

### Patient Samples

All patient samples obtained were de-identified to protect patient confidentiality and had Georgetown University IRB-approval and consent. All the tissue samples were obtained from GU/LCCC Histopathology & Tissue Shared Resource (http://lombardi.georgetown.edu/research/sharedresources/htsr/) and written informed consent was obtained from all the participants for urine sample. Briefly, 40 formalin-fixed, paraffin-embedded (FFPE) tissue specimen blocks from radical prostatectomy consisting 15 Caucasian American (CA) and 25 African American (AA) were obtained from Lombardi Histopathology and Tissue Shared Resource (HTSR) between 1997–2002. Urine samples from 36 PCa patients (18 CA and 18 AA) and 12 age and ethnicity matched healthy donors (6 CA and 6 AA) were obtained from Georgetown University Hospital Cyberknife Prostate Cancer Program between 2009 to 2012.

### Laser Capture Microdissection (LCM)

Hematoxylin and eosin (H&E) stained slides from archival FFPE blocks were reviewed by a board certified pathologist, for the identification of PCa foci as well as adjacent normal tissue. LCM was performed on Arcturus laser capture microscope system with 5,000 to 6,000 laser pulses for each sample to capture 30,000 to 50,000 cells of PCa foci and adjacent normal tissue. Captured cells were immediately frozen at −80°C until further use.

### RNA Extraction and miRNA Expression Profiling

RNA extraction from microdissected cells and urine samples was performed using Recover All™ Total Nucleic Acid Isolation and mirVana™ miRNA isolation kits respectively (Ambion, Austin, TX) as per the manufacturer’s protocol. RNA was quantified using NanoDrop ND-1000 Spectrophotometer (Thermo Scientific, Waltham, MA) and Agilent 2100 Bioanalyzer (Santa Clara, CA).

RNA was converted to cDNA using Megaplex™ Primer pools and Taqman miRNA Reverse Transcription Kit (Applied Biosytems, Grand Island, NY). A comprehensive miRNA expression profiling was carried out using TaqMan Array Human MicroRNA A Card v2.0 following manufacturer’s recommendations (Applied Biosytems, Grand Island, NY). Quantitative real-time PCR (qRT-PCR) was carried out to screen a total of 377 unique human miRNAs by Applied Biosystems 7900 HT Fast real-time PCR sequence detection system. Data was analyzed on sequence detection system (SDS) software (version 2.3, Applied Biosytems, Grand Island, NY). Relative miRNA expression levels were normalized against endogenous control U6 snRNA.

### Validation by qRT-PCR

Expression levels of selected miRNAs were measured in 40 PCa patients using inventoried TaqMan miRNA Assays (Applied Biosytems, Grand Island, NY) following manufacturer’s recommendations, on 7300 Real-Time PCR System (Applied Biosytems, Grand Island, NY). Briefly, 10 ng of RNA was reverse transcribed using specific stem-loop primers. Tissue samples were normalized to internal standard control U6 snRNA whereas, RNU48 was used as normalizing control for urine samples. Non reverse transcriptase (RT) controls were used to rule out the possibility of potential genomic DNA contamination. MicroRNAs with threshold cycle (Ct) values of ≥38 were excluded from the analysis. All samples underwent reverse transcription and qRT-PCR simultaneously to minimize errors introduced by variations in reaction efficiency.

### Data Mining, Target Identification and Pathway Analysis

Expression of selected miRNAs were analyzed in Gene Expression Omnibus (GEO) database in ‘R’ by GEO2R [21]. The mRNA targets for differentially expressed miRNAs were identified using online software and databases such as TargetScan [22], PicTar [23] and miRDB [24] followed by additional experimentally verified targets from miRTarBase [25]. Possible gene-gene interactions and functional clustering among targets of miRNAs, was performed using Ariadne Pathway Studio 9.0.

### Statistical Analysis

The raw human MicroRNA-A Card v2.0 array data was statistically analyzed by Integromics RealTime StatMinier software version 4.0, and R/Bioconductor software version 2.9.2. The 2^−ΔΔCt^ method [26] was employed for pre-processing and fold change calculations. Differentially expressed miRNAs between PCa tissues and adjacent normal tissue were identified using Limma package [27] which employs the empirical Bayesian model to deal with the small sample size compared to the relatively much larger number of miRNAs. The *p-*values were adjusted using Benjamin–Hochberg false discovery rate (FDR) correction [28].

All qRT–PCR experiments were conducted according to the MIQE (minimum information for publication of quantitative realtime PCR experiments) guidelines [29]. Each amplification reaction was performed in triplicate, and mean value of the three-cycle threshold was used for further analysis. Data are presented as means ± SE and P value≤0.05 was considered statistically significant. The nonparametric Student’s T-test was used for comparing two groups (cancer vs. non-cancer), and all statistics were adjusted using the Holm-Bonferonni correction for multiple comparisons. All the box plots represent miRNA levels relative to U6 snRNA, transformed into quantities using the formula 2^−ΔCt^ [30].

Receiver operating characteristic (ROC) curves were constructed and area under curve (AUC) was estimated to study the feasibility of using the particular miRNA to discriminate PCa patients from healthy controls. Logistic regression was used to construct ROC curves using miRNA expression levels. All the statistical analysis was performed using GraphPad Prism v6 (La Jolla, CA).

## Results

### Expression Profiling of miRNAs in Prostate Cancer Tissues

Assessing changes in miRNA expression in PCa tissues and bio-fluids offer a promising tool for identifying specific biomarkers that can aid in the diagnosis and prognosis of PCa. Initially, we used laser-captured microdissection (LCM) to capture region of cancer tissues and the corresponding adjacent normal counterparts from FFPE samples of radical prostatectomy patients. MicroRNA expression profiling was performed in 10 PCa patients with Gleason (GS) 6–7 (5 AA and 5 CA). The cancer and adjacent normal regions were identified by the pathologist BVSK. Captured cells from various cancer regions from each patient were pooled to reflect heterogeneity of PCa and patient population; adjacent normal tissue counterparts served as non-cancer control. We performed miRNA profiling analysis and found that majority of miRNAs were downregulated in cancer tissues, with the exception of miR-367, miR-758 and miR-190 which were found to be upregulated (Table 1). For validation, we selected the 3 upregulated miRNAs (miR-367, miR-758 and miR-190) and 5 downregulated miRNAs (miR-205, miR-214, miR-212, miR-221 and miR-99b) based on their published role in cancer biology. Profiling data for all the 10 patients (5 CA and 5 AA) has been submitted to GEO database (accession number GSE48430).

**Table 1:** Differentially expressed miRNAs in Prostate Cancer Tissues.

miRNA	Chromosome Location	FDR	Fold Change	p value
Up Regulated				
hsa-miR-367*	4q25	1.85E-03	245.6	7.82E-05
hsa-miR-758*	14q32.31	0.64151	9.1	2.58E-02
hsa-miR-190*	15q22.2	0.64151	5.7	2.82E-02
hsa-miR-221*	Xp11.3	2.45E-03	−155.1	1.10E-04
hsa-miR-205*	1q32.2	7.49E-02	−126.3	8.93E-03
hsa-miR-212*	17p13.3	1.85E-03	−74.8	7.85E-05
hsa-miR-99b*	19q13.41	7.66E-02	−7.8	9.75E-03
hsa-miR-214*	1q24.3	0.216707	−5.3	4.60E-02
hsa-miR-203	14q32.33	0.132043	−16.4	2.21E-02
hsa-miR-127-3p	14q32.2	2.75E-02	−15	2.33E-03
hsa-miR-130a	11q12.1	0.156529	−13	2.95E-02
hsa-miR-335	7q32.2	0.138395	−12.7	2.42E-02
hsa-miR-376	14q32	0.127945	−10.1	2.10E-02
hsa-miR-10a	17q21.32	0.177871	−9.8	3.54E-02
hsa-miR-589	7p22.1	0.336638	−9.4	0.03643
hsa-miR-422a	15q22.31	0.336638	−8.4	8.40E-03
hsa-miR-10b	2q31.1	0.155758	−20.7	2.85E-02
hsa-miR-25	7q22.1	0.144053	−5.6	2.56E-02
hsa-miR-210	11p15.5	8.10E-02	−37.3	1.05E-02
hsa-miR-99a	21q21.1	0.138395	−5.2	2.41E-02
hsa-miR-429	1p36.33	0.216707	−4.9	4.57E-02
hsa-miR-92a	13q31.3	0.179541	−4.7	3.62E-02
hsa-miR-100	11q24.1	0.336638	−3.1	2.14E-02
hsa-miR-222	Xp11.3	0.336638	−2.3	1.72E-02
hsa-miR-484	16p13.11	0.336638	−2.3	3.49E-02
hsa-miR-125b	11q23	0.336638	−2.2	1.76E-02
hsa-miR-574-3p	4p14	0.336638	−2	1.72E-02
hsa-miR-328	16q22.1	0.336638	−2	2.48E-02
hsa-miR-483-5p	11p15.5	0.336638	−1.8	1.19E-02
hsa-miR-331-3p	12q22	0.336638	−1.8	1.56E-02
hsa-let-7c	21q21.1	0.336638	−1.6	1.09E-02
hsa-miR-135a	3p21.1	0.089442	−17.2	1.40E-02

* miRNAs selected for tissue validation.

### Validation of miRNAs by Quantitative RT-PCR

**Figure 1:**
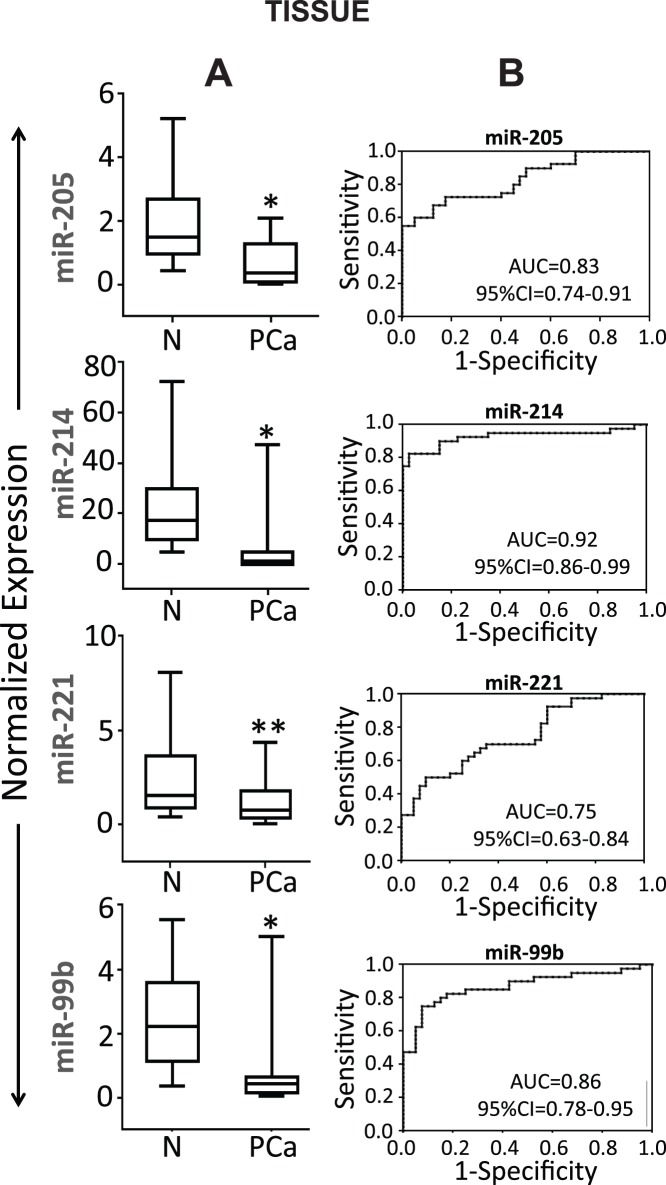
Expression of selected miRNAs in tissue samples. (A) Validation of miRNAs by qRT-PCR: Box plots representing the tissue expression level of four miRNAs in 40 FFPE PCa tissues and their paired adjacent normal tissues. Expression levels of the miRNAs are normalized to U6 snRNA as endogenous control. Statistically significant differences were determined using unpaired Student’s T-test. We detected significant decrease in the expression of miR-205 (*p*<0.0001), miR-214 (*p*<0.0001), miR-221 (*p*<0.001) and miR-99b (*p*<0.0001) in PCa tissues as compared to adjacent normal tissues. *denotes *p*<0.0001 and **denotes *p*<0.001. (B) Receiver operating characteristic (ROC) curve analysis: ROC curve for four miRNAs were made to differentiate PCa from healthy tissues. The area under the ROC curve (AUC) for each miRNA conveys its accuracy for differentiation of PCa tissues and normal tissues in terms of sensitivity and specificity.

The selected 8 miRNAs were validated in LCM-dissected tumor and adjacent normal tissue from 40 patients including the 10 patients used for miRNA profiling. Table 2 shows clinico-pathological characteristics of the patients. The qRT-PCR results of 40 patient sample (15 CA and 25 AA) specimens revealed decreased expression of miR-205 (*p*<0.0001), miR-214 (*p*<0.0001), miR-221 (*p*<0.001) and miR-99b (*p*<0.0001) in cancer tissue as compared to adjacent non-cancer tissue (Fig. 1A). Although miR-212 showed a trend towards downregulation in PCa as compared to adjacent normal tissue, the difference was not statistically significant (*p* = 0.061). There was no significant difference in the relative expression of miR-758, and miR-190 in tumor tissue compared to their adjacent normal counterparts and the levels of miR-367 could not be detected after 38 cycles (data not shown). Therefore, miR-212, miR-758, miR-190 and miR-367 were excluded from further study.

**Table 2:** Clinical-pathological characteristics of the prostate cancer patients.

Characteristics	CaucasianAmerican	AfricanAmerican
Number of patients, n (%)	15 (37.5)	25 (62.5)
Age at diagnosis		
Mean ± SD (years)	59.2±5.6	59.5±5.7
≤65, n (%)	13 (86.6)	20 (80.0)
>65, n (%)	03 (13.3)	05 (20.0)
Stage, n (%)		
I–II	12 (80.0)	21 (84.0)
III–IV	03 (20.0)	04 (16.0)
Primary tumor, n (%)		
pT1–pT2	12 (80.0)	21 (84.0)
pT3–pT4	03 (20.0)	04 (16.0)
Lymph node metastasis, n (%)		
N0	15 (100.0)	23 (92.0)
N1–N4	00 (0.0)	02 (8.0)
Gleason sum, n (%)		
≤6	7 (46.0)	10 (40.0)
7	6 (40.0)	12 (48.0)
≥8	2 (13.0)	03 (12.0)

Receiver operating characteristics (ROC) curves were constructed to explore sensitivity and specificity of miR-205, miR-214, miR-221 and miR-99b and assess their potential to be used as biomarker to discriminate between PCa and disease free individuals (Fig. 1B). The results suggests that the four miRNAs can discriminate between the two groups with high precision; miR-205, AUC = 0.83 (95% CI = 0.74–0.91); miR-214 AUC = 0.92 (95% CI = 0.86–0.99); miR-221 AUC = 0.75 (95% CI = 0.63–0.84) miR-99b AUC = 0.86 (95% CI = 0.78–0.95) (Fig. 1B).

### Expression of miRNAs in GEO Datasets

**Figure 2:**
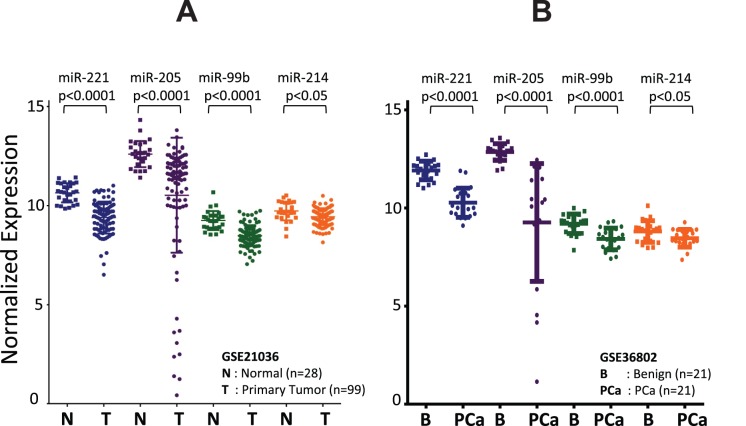
Expression level of four miRNAs in PCa based on Gene Expression Omnibus (GEO) database. microRNA validation data for (A) 99 primary PCa patients and 28 normal controls and (B) 21 patients each with clinically localized primary PCa and benign PCa were obtained from the NCBI GEO database (GEO accession no. GSE21036 and GSE36802, respectively). Shown are the scatter plots of expression level of miR-205, miR-214, miR-221 and miR-99b in the datasets obtained from GEO database.

Data mining was performed using GEO2R to check the status of differentially expressed miRNAs in publically available GEO datasets. Microarray datasets GSE21036 (Taylor et al.) [31] and GSE36802 (Lin et al.) [32] were queried for the expression of selected four miRNAs (miR-205, miR-214, miR-221 and miR-99b) of interest. Supporting our tissue data, in GSE21036, all the four miRNAs were also downregulated in primary prostate tumors (n = 99) as compared to normal prostate tissues (n = 28) (miR-221, *p*<0.0001; miR-205, *p*<0.0001; miR-99b, *p*<0.0001; miR-214, *p*<0.05). Similarly, in GSE36802, we observed downregulation of all the four miRNAs in clinically localized primary tumor (n = 21) when compared to benign PCa (n = 21) (miR-221, *p*<0.0001; miR-205, *p*<0.0001; miR-99b, *p*<0.0001; miR-214, *p*<0.05) (Fig. 2).

### Pathway Network Analysis of Differentially Modulated miRNAs

**Figure 3:**
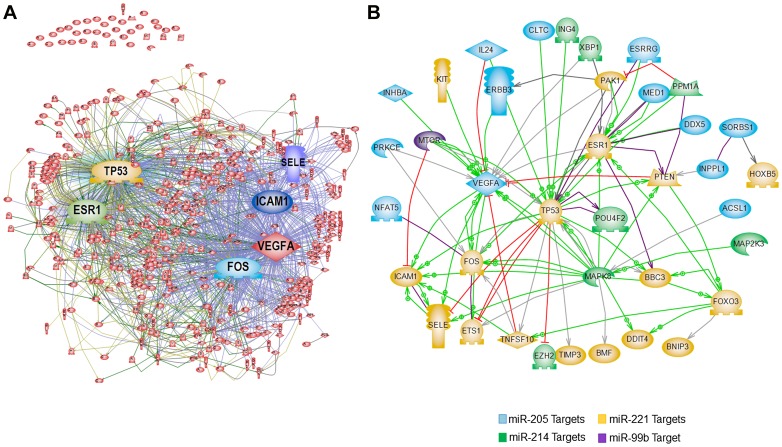
Pathway network of the genes targeted by differentially modulated miRNAs. Pathway network was constructed for commonly predicted target mRNAs of differentially modulated miRNAs (miR-205, miR-214, miR-221 and miR99b), by using Pathway Studio 9.0 (A) Commonly predicted miRNA targets with common regulators. (B) Directly interacting targets.

To gain functional insights into the role of miRNA in PCa and to investigate the possible gene-gene interactions between targets of the four miRNAs, we constructed an interaction network using Pathway Studio 9.0. A total of 98 mRNA targets were identified by (a) common targets obtained from three softwares (TargetScan, PicTar and miRDB) for all the 4 miRNAs and (b) validated targets for the 4 miRNAs were obtained by miRTarBase. We added common regulators along with direct interactions–direct regulation of gene expression, protein-protein binding, or promoter binding. A signaling complex evolved from 62 of the 98 targets revealed VEGFA, ICAM1, p53, ESR1, SELE and FOS as common nodes/hubs of several interactions suggesting commonly driven pathways by the connected genes and candidate for future experimental verification and functional studies (Fig. 3). The rest 36 targets were excluded by the software due to lack of common connectors. A second pathway, constructed to identify miRNA targets with direct interactions, suggested similar nodes/hubs (Fig. 3).

### Ethnic Variation in miRNA Expression

**Figure 4:**
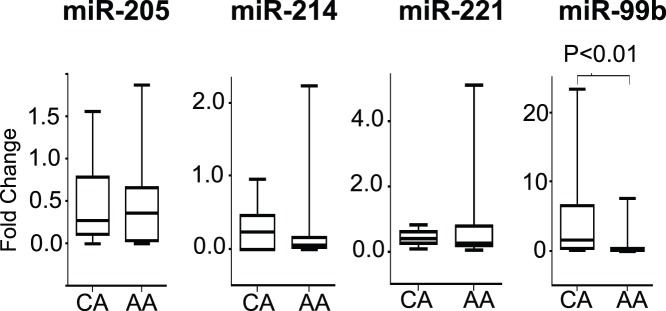
Comparison of miRNA expression on the basis of ethnicity. Box plots represent the fold difference in expression levels of four miRNAs in PCa tissues as compared to their normal adjacent counterpart. Data for 15 Caucasian American (CA) and 25 African American (AA) patients as assessed by qRT-PCR is shown. Expression levels of the miRNAs were normalized to U6 snRNA as endogenous control. Statistically significant differences were determined using unpaired Student’s T-test.

African American **(**AA) men have a higher incidence of PCa as compared to Caucasian American (CA) men. We asked whether expression levels of miR-205, miR-214, miR-221 and miR-99b were different between the two populations (n = 15 CA; n = 25 AA). To test this we compared the fold differences of each miRNA in cancer tissue relative to their adjacent normal tissue in AA and CA samples. As shown in Fig. 4, there was no significant difference in expression patterns of miR-205, miR-214, and miR-221 between CA and AA population. However, a significantly decreased expression (*p*<0.01) of miR-99b was observed in cancer tissue from AA PCa patients when compared to CA PCa patients (Fig. 4).

### Detection of miRNAs in Urine Samples

**Figure 5:**
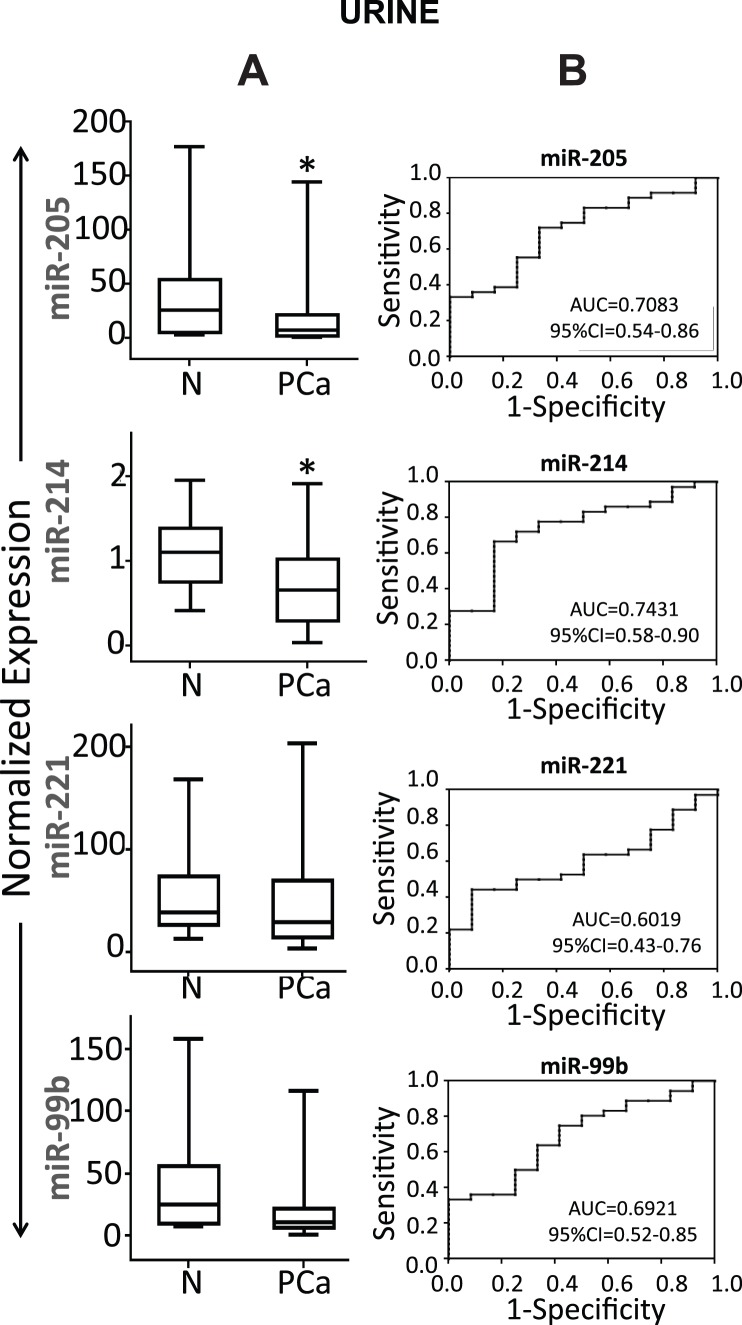
Identification of selected miRNAs in Urine. (A) Box plots representing the urine expression level of four miRNAs (miR-205, miR-214, miR-221 and miR-99b) in urine from 36 PCa patients and 12 healthy individuals as assessed by qRT-PCR. Expression levels of the miRNAs are normalized to RNU48 as endogenous control. Statistically significant differences were determined using Student’s T-test. We detected significant decreased expression of miR-205 (*p*<0.05), miR-214 (*p*<0.05) in urine of PCa patients as compared to healthy controls. No significant difference in expression levels of miR-221 and miR-99b were observed. *denotes *p*<0.05. (B) Receiver operating characteristic (ROC) curve analysis of four miRNAs was used to differentiate PCa patients from healthy individuals. The area under the ROC curve (AUC) for each miRNA conveys its accuracy for differentiation of PCa patients and healthy subjects in terms of sensitivity and specificity.

**Figure 6:**
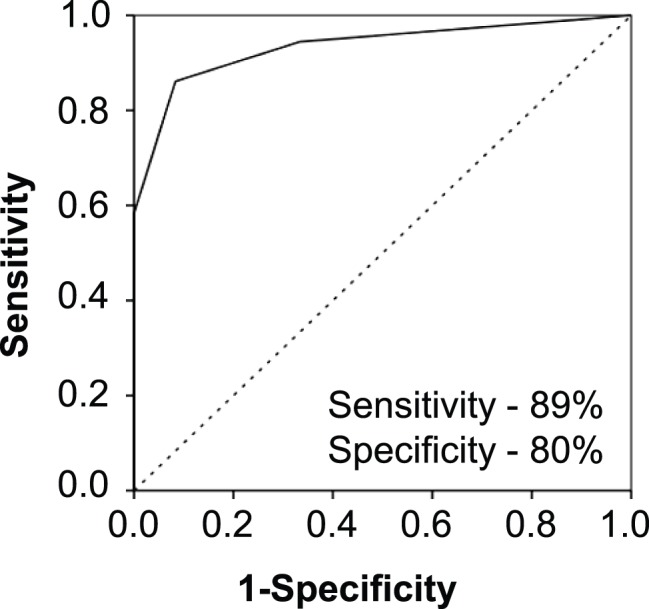
Prognostic accuracy of miR-205 and miR-214 in urine samples. The quantification of miR-205 and miR-214 together can enhance the diagnostic value and can be used to discriminate PCa patients from healthy individuals with 89% sensitivity and 80% specificity.

Tissue biopsies are invasive and not the preferred source for biomarkers. We next explored the possibility, whether miR-205, miR-214, miR-221, and miR-99b could be detected non-invasively by analyzing urine samples from PCa patients. From an ongoing study, we selected 36 PCa patients and 12 age and ethnicity matched healthy donors as a non-cancer control group. Table 3 shows characteristics of PCa patients and healthy individuals recruited in the study for urine samples. Since for miRNA profiling we used tissue specimens with GS6 and GS7, we obtained urine samples from patients with GS6 and GS7 as a reflection of the tissue specimens. All four miRNAs (miR-205, miR-214, miR-221 and miR-99b) were present in detectable concentration in urine samples. We found that urinary miR-205 (*p*<0.05) and miR-214 (*p*<0.05) levels were significantly lower in the cancer group as compared to healthy control group (Fig. 5A). No significant difference in the expression levels of miR-221 and miR-99b were observed. To evaluate the diagnostic potential, ROC curves for all 4 miRNAs analyzed in the urine samples were constructed. The ROC curve showed that miR-205 and miR-214 can discriminate PCa patients from normal control with AUC: 0.7083 (95% CI = 0.54–0.86) and 0.7431 (95% CI = 0.58–0.90), respectively (Fig. 5B). Also, miR-205 and miR-214 if employed together can discriminate the PCa patients from healthy individuals with 89% sensitivity and 80% specificity (Fig. 6). Taken together, our results show that miR-205 and miR-214 are present in both tissue and urine of PCa patients, suggesting that urine could be employed to detect changes in the expression levels of miRNAs.

**Table 3:** Demographic and clinico-pathological characteristics of the participants for urine samples.

Characteristics	Caucasian American		African American	
	Patients	Normal	Patient	Normal
Number of patients, n (%)	18(50.0)	6(50.0)	18(50.0)	6(50.0)
D’Amico Risk Group, n (%)				
Low Risk	6(33.3)	–	6(33.3)	–
Medium Risk	6(33.3)	–	6(33.3)	–
Age				
Mean±SD (years)	66.8(5.9)	67(7.9)	66.2(6.0)	67.6(5.8)
≤65, n (%)	6(33.3)	3(50.0)	8(44.4)	2(33.3)
>65, n (%)	12(66.6)	3(50.0)	10(55.5)	4(66.6)
Primary tumor, n (%)				
pT1–pT2	18(100.0)	–	17(94.4)	–
pT3–pT4	0(0)	–	1 (5.5)	–
Lymph node metastasis, n (%)				
N	14(77.7)	–	15(83.3)	–
N0	4(22.2)	–	3(16.6)	–
N1–N4	0(0)	–	0(0)	–
Distant metastasis, n (%)				
Mx	14(77.7)	–	11(61.1)	–
M0	4(22.2)	–	7(38.8)	–
M1	0(0)	–	0 (0)	–
Gleason sum, n (%)				
≤6	7(38.8)	–	8(44.4)	–
7	6(33.3)	–	7(38.8)	–
PSA levels (ng/ml)				
Mean (± SD)	12.0(18.3)	1.9(0.89)	13.7(22.1)	1.1(0.95)
<6, n (%)	9(50.0)	6(100.0)	7(38.8)	6(100)
≥6, n (%)	9(50.0)	0(0)	11 (61.1)	0(0)

## Discussion

Alterations in miRNA expression levels, resulting in the modulation of multiple signaling pathways, have been linked to initiation and progression of PCa. In the present study, using global miRNA profiling followed by validation studies, we showed that miR-205, miR-214, miR-221 and miR-99b were significantly downregulated in PCa tissues as compared to adjacent normal tissue counterparts.

One of the most studied microRNA, miR-205 which is shown to be down regulated in various cancers including PCa [33]–[34], was also identified as downregulated miRNA in PCa in our study. miR-205 has been shown to be epigenetically repressed tumor suppressor in PCa [35] that exerts its tumor-suppressive functions in the human prostate through down-regulation of multiple targets such as BCL2 [36], protein kinase C epsilon [34] and androgen receptor (AR) [37]. The loss of miR-205 has also been associated with poor prognosis, apoptosis resistance in PCa and is suggested to be a hallmark of epithelial-mesenchymal transition [37]–[41]. Other expression array studies have also identified miR-205 being repressed in PCa [33]–[34], [42]–[43]. Low-level expression of miR-205 is also a prognostic marker of human head and neck squamous cell carcinoma [44] and the locus has been reported to be silenced by promoter hypermethylation in invasive bladder tumors [45]. Present study as well as previous literature affirms an important role of miR-205.

The next aberrantly expressed miRNA in our study was miR-214 which has been shown to be frequently downregulated in cervical cancer [46], ovarian cancer [47]–[48], hepatocellular carcinoma [49]–[51] and cholangiocarcinoma [52]. In contrast, high expression of miR-214 has been associated with pancreatic cancer [53] and with unfavorable outcome in overall survival in gastric carcinoma [54]. MiR-214 deregulation has not previously been reported in prostate cancer. This is the first study to show that miR-214 is aberrantly expressed in PCa. The significant down- regulation in tissue biopsies as well as urine specimens from PCa patients introduces miR-214 as a novel player in PCa, which needs further exploration.

Another downregulated miRNA in our study was miR-221. Importantly, miR-221 was identified as the most significantly modulated miRNA in the two PCa GEO datasets. MiR-221 is de-regulated in variety of cancers, primarily as an over-expressed miRNA [55]–[59].

In PCa, downregulation of miR-221 has been reported in TMPRSS2: ERG fusion-positive PCa and is significantly associated with metastasis and biochemical recurrence [58], [60]. Few studies have also reported an oncogenic role of miR-221 in PCa [61]–[62] and the development and maintenance of castrate resistance phenotype [63]–[64]. Our result is one of the few reporting down-regulation of miR-221 in PCa tissues and warrant further studies.

Another very important miRNA identified in our study was miR-99b. The miR-99 family including miR-99b has been associated with PCa suppression and prognosis [63]. Down-regulation of miR-99b has also been observed in patients with lung cancer [65]. Mir-99b has also been shown to be over-expressed in synovial sarcoma [66] and associated with the presence of lymph node metastasis in esophageal cancer [67]. Our results indicate that down-regulation of miR-99b is more pronounced in AA PCa tissues as compared to CA PCa tissues (Fig. 4). Functional studies on miR-99b are limited and these novel observations demand further investigations into the role of miR-99b targets. Taken together, present data indicates that, along with various other factors, expression variation of miRNAs such as miR-99b can explain the ethnic aggressiveness of the disease.

To explore the signaling networks regulated by these four miRNAs, we constructed a pathway of their common predicted targets using available bioinformatics tools and validated targets from the literature. Adding the common regulators, we identified central signaling hubs that have been reported to play an important role in cancer cell signaling suggesting an important role of these four miRNAs in PCa (Fig. 3). Next, we constructed a direct interacting network of miRNA targets without common regulators. VEGFA, p53, ESR1, FOS and ICAM1 remained as central hubs [68]–[72]. The mTOR was the only miR-99b target that was included in the common network. We identified miR-99b as differentially modulated miRNA in AA PCa. mTOR pathway plays an important role in PCa and has been associated with an aggressive disease, resistance to therapy and development of castrate resistant PCa [73]. mTOR inhibitors have also been evaluated as therapy for CRPC [74]. Association of miR-99b with AA PCa is highly significant and further studies in our lab are directed towards characterizing miR-99b and its targets in rendering aggressive PCa phenotype.

The concept of using urine for the detection of differentially expressed miRNAs as biomarker is relatively new. Considering the idea that tissue derived expression patterns of miRNAs may help us to assess circulating miRNAs as biomarkers for various types of cancers, most promising miRNAs observed in our study were studied in urine of PCa patients to evaluate their diagnostic or prognostic potential. Consistent with our findings in tissue samples, we were also able to detect miR-205, miR-214, miR-99b and miR-221 in the urine samples of PCa patients. The levels of miR-205 and miR-214 were significantly low in PCa patients and can be explored as a non-invasive diagnostic biomarker for PCa. Although the sensitivity and specificity is greater in tissue, urinary miR-205 and miR-214 levels together can discriminate patients from healthy individuals with high precision. To our knowledge, our study reveals for the first time that miR-205 and miR-214 can provide an alternative non-invasive modality to distinguish between PCa and healthy individuals and can serve as a reliable biomarker for PCa.

The use of urine as a specimen for tumor marker remains challenging in view of the fact that urine contains a wide variety of biomolecules including high amount of nucleases and RNases. However, due to the small size, miRNAs are more stable against RNase degradation which advocates their merit as biomarkers [75]. Few studies have examined the potential of urinary miRNAs as diagnostic and prognostic markers of bladder cancer, kidney disease, urothelial cancer, hepatocellular carcinoma and renal cell carcinoma [76]–[80]. Although miRNA levels have been studied in various diseases, no comprehensive study for circulating miRNA in urine for PCa has been reported so far, with the exception of Bryant et al [81]–[82]. Significantly higher concentration of miR-107 and miR-574-3p were quantified in the urine of men with PCa compared to controls [82]. To the best of our knowledge, we demonstrate for the first time, that miR-205 and miR-214 are downregulated in PCa tissue as well as in urine from PCa patients. We acknowledge the limitation of our study that the tissues and urine obtained were not from the same patients and our small sample size. Nevertheless, our study provides proof of concept that deregulated miRNAs in tissues can be explored as non-invasive urine biomarkers in PCa. The ROC curve demonstrates that miR-205 and miR-214 together have a capability to distinguish between healthy individuals and PCa patients with 89% sensitivity and 80% specificity. The present finding emphasizes the role of miR-205 and miR-214 in PCa and bears the prospective to serve as good molecular markers helping to identify PCa patients. Further investigations are needed in a larger cohort of patients to strengthen these finding and use them as noninvasive screening tool in combination with routine clinical tests. Also, understanding of the biological functions of these miRNAs in patients with PCa will significantly help in the development of new targeted drugs and will facilitate in appropriate clinical management.
